# *IceBear*: an intuitive and versatile web application for research-data tracking from crystallization experiment to PDB deposition

**DOI:** 10.1107/S2059798320015223

**Published:** 2021-01-26

**Authors:** Ed Daniel, Mirko M. Maksimainen, Neil Smith, Ville Ratas, Ekaterina Biterova, Sudarshan N. Murthy, M. Tanvir Rahman, Tiila-Riikka Kiema, Shruthi Sridhar, Gabriele Cordara, Subhadra Dalwani, Rajaram Venkatesan, Jaime Prilusky, Orly Dym, Lari Lehtiö, M. Kristian Koski, Alun W. Ashton, Joel L. Sussman, Rik K. Wierenga

**Affiliations:** a Biocenter Oulu, University of Oulu, Oulu, Finland; b Faculty of Biochemistry and Molecular Medicine, University of Oulu, Oulu, Finland; c Diamond Light Source, Harwell Science and Innovation Campus, Didcot, United Kingdom; d Bioinformatics and Biological Computing Unit, Life Science Core Facility, Weizmann Institute of Science, Rehovot 7610001, Israel; e Israel Structural Proteomics Center, Life Science Core Facility, Weizmann Institute of Science, Rehovot 7610001, Israel; f Department of Structural Biology, Weizmann Institute of Science, Rehovot 7610001, Israel

**Keywords:** research-data management, crystallization, X-ray data collection, ISPyB, metadata, *IceBear*

## Abstract

The web-based *IceBear* software is a versatile tool to monitor the results of crystallization experiments and is designed to facilitate supervisor and student communications. It also records and tracks all relevant information from crystallization setup to PDB deposition in protein crystallography projects. Fully automated data collection is now possible at several synchrotrons, which means that the number of samples tested at the synchrotron is currently increasing rapidly. Therefore, the protein crystallography research communities at the University of Oulu, Weizmann Institute of Science and Diamond Light Source have joined forces to automate the uploading of sample metadata to the synchrotron. In *IceBear*, each crystal selected for data collection is given a unique sample name and a crystal page is generated. Subsequently, the metadata required for data collection are uploaded directly to the ISPyB synchrotron database by a shipment module, and for each sample a link to the relevant ISPyB page is stored. *IceBear* allows notes to be made for each sample during cryocooling treatment and during data collection, as well as in later steps of the structure determination. Protocols are also available to aid the recycling of pins, pucks and dewars when the dewar returns from the synchrotron. The *IceBear* database is organized around projects, and project members can easily access the crystallization and diffraction metadata for each sample, as well as any additional information that has been provided via the notes. The crystal page for each sample connects the crystallization, diffraction and structural information by providing links to the *IceBear* drop-viewer page and to the ISPyB data-collection page, as well as to the structure deposited in the Protein Data Bank.

## Abstract 2

The *IceBear* web application for monitoring and recording the results of crystallization experiments is introduced. This software includes tools for interacting directly with the ISPyB synchrotron database: metadata from shipped crystals can be uploaded directly to ISPyB, and for each sample a link to the synchrotron ISPyB diffraction information becomes available in *IceBear*.

## 1. Introduction   

Data collection at synchrotrons has generally become a routine step in the protein structure-determination pipeline (Helliwell, 2017 ▸; Dauter & Wlodawer, 2016 ▸; Owen *et al.*, 2016 ▸; Grimes *et al.*, 2018 ▸) that starts with crystallization of the protein and results in the deposition of its structure and experimental structure factors in the Protein Data Bank (PDB). In Europe, the Diamond Light Source (DLS) in the UK and the European Synchrotron Radiation Facility (ESRF) in France have developed powerful data-processing pipelines by which the collected data can be processed automatically (Monaco *et al.*, 2013 ▸; Materlik *et al.*, 2015 ▸). Downstream processing pipelines are also available for automatic determination of the structure and the mode of binding of ligands (Thomas *et al.*, 2019 ▸; Förster & Schulze-Briese, 2019 ▸), if the relevant metadata such as sequence, model structure and ligand are available. Efficient use of this impressive technology by home laboratories typically located on campuses of universities, research institutes or (biotech) industries, on sites physically far away from synchrotron sites, is very important. This requires easy access to the crystallization data as well as to the diffraction information and any other relevant information related to the crystal treatment, not only for the researcher preparing the crystals but also for each of the members of the project team.

**Figure 1:**
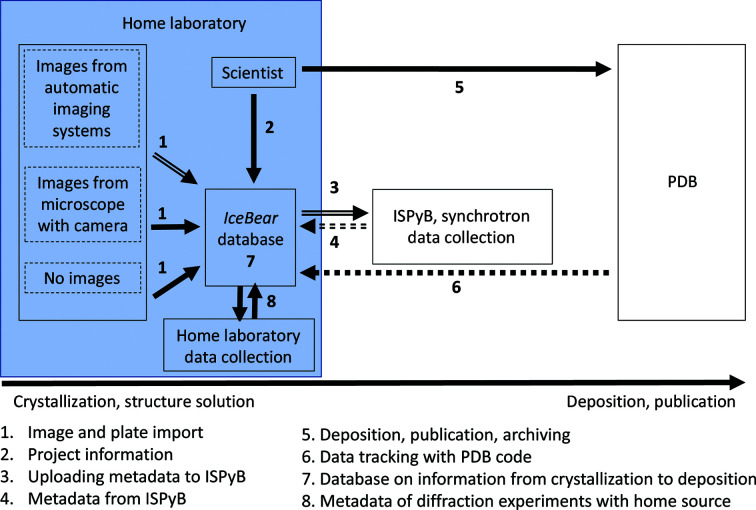
Flow of the metadata of the structure-determination pipeline from crystallization to publication and deposition. *IceBear* assigns selected crystals a unique sample name and the ISPyB diffraction information is associated with the corresponding sample name. Uploading the metadata from the home laboratory to ISPyB is performed with the *IceBear* shipment module, as described in the text. The scheme visualizes the integration of the ISPyB diffraction metadata in the structure-determination pipeline when data are collected at a synchrotron running ISPyB. Diffraction metadata of selected crystals using other X-ray facilities, for example a home source, for crystal testing as well as data collection can also be uploaded into the database. Solid arrow: metadata are transferred manually. Double arrow: metadata are transferred automatically. Double-dashed arrow: hypernet links are transferred automatically. Dotted arrow: the *IceBear* database can be searched using the PDB code of a structure.

Crystallization is the critical first step of the structure-determination pipeline (Lynch *et al.*, 2020 ▸; Abrahams & Newman, 2019 ▸; Rosa *et al.*, 2020 ▸; Mayo *et al.*, 2005 ▸). The workflow shown schematically in Fig. 1 ▸ visualizes the very common situation in which structural biology research groups solve and refine their structures using synchrotron data sets collected from crystals grown in their home laboratories. Usually, a wide range of crystallization screens are used in crystallization campaigns to maximize the chance that crystals will be obtained. This requires the monitoring of thousands of crystallization drops. Typically, commercially available software for imaging systems is used for this. In large crystallization facilities, more comprehensive laboratory information-management systems (LIMS) have been developed for this purpose, such as *xtalPiMS* (Daniel *et al.*, 2011 ▸) and *CRIMS* (Dupeux *et al.*, 2011 ▸). In addition, to address the needs of crystallization home laboratories, software has been developed by the University of Oulu and DLS, which is referred to as *IceBear* (*Integrated Crystal-data-tracking Enhancing Bio­chemistry Education And Research*; https://www.youtube.com/watch?v=ZuJhQNUzn5E). Once suitable crystals have been obtained then these crystals need to be harvested, used for ligand-binding studies, cryocooled and shipped in dewars to a synchrotron. The technical procedures for shipping dewars to synchrotrons have been well worked out; however, there are no robust data-management tools available to record and preserve the metadata of the crystallization and crystal-treatment protocols and to connect these metadata to the diffraction information. A large number of samples are typically handled at the synchrotron during one session, which makes data handling and archiving very challenging. In order to address these needs, the protein crystallography communities at the University of Oulu in Finland and the Weizmann Institute of Science in Israel have joined forces together with DLS, and have extended *IceBear* with several modules that make it possible to upload the metadata directly to ISPyB (Fig. 1 ▸). More generally, this facilitates data tracking from crystallization to PDB deposition. The development of this software has benefitted from previous work and experience with the *xtalPiMS* software (Daniel *et al.*, 2011 ▸), as well as from the *eHTPX* (Berry *et al.*, 2006 ▸; Allen *et al.*, 2003 ▸) and *CRIMS* (De Maria Antolinos *et al.*, 2015 ▸) initiatives to exchange metadata with ISPyB at DLS and ESRF, respectively.

**Figure 2:**
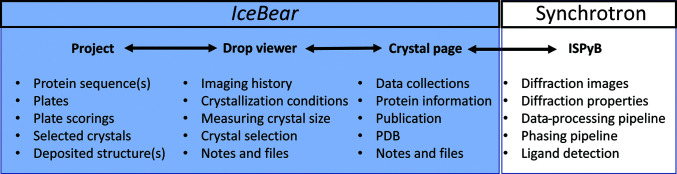
Flow of the metadata between key modules within *IceBear* and between *IceBear* and ISPyB. The diffraction information in ISPyB is accessible via links that are available on the crystal page. The listed items below the module names concern information that is available on the pages of the respective module. Data tracking to monitor the progress of a project is facilitated by having clickable links between the modules and by being able to search in the *IceBear* database for plates, pins, pucks, dewars, sample names and PDB codes, as explained in the text.

The ISPyB database (Fisher *et al.*, 2015 ▸; Delagenière *et al.*, 2011 ▸; De Maria Antolinos *et al.*, 2015 ▸) is an essential tool for guiding the diffraction experiments at the beamline and also for handling the diffraction information collected at the synchrotrons. It is in use by all of the synchrotrons in Europe (Oscarsson *et al.*, 2019 ▸). Once protein crystals have been obtained, these crystals need to be harvested, mounted on pins, cryocooled, possibly tested with a home-source X-ray generator and stored in dewars, which are then shipped to the synchrotron for high-resolution X-ray data collection. Simultaneously, metadata for each crystal must be uploaded to the ISPyB database. The information gathered in ISPyB remains available to the users indefinitely, and therefore is an invaluable information resource for structural biology projects that use synchrotrons for data collection. In home laboratories that are an integral part of universities, crystals are typically very often generated by students who are not yet well trained in protein crystallography. Supervisor–student interactions are essential to make sure that the important metadata on crystallization are well documented and to make sure that data collection and data processing are successfully carried out. Nowadays tens or hundreds of crystals for a particular project are routinely shipped to European synchrotrons, so a user-friendly IT tool to help the novice as well as the experienced user with data tracking becomes essential. Such an IT tool should guide the researcher to provide all of the important information on the crystallization experiment and should make this information easily available to the project members, together with the diffraction information collected in the ISPyB database. This is even more relevant now that DLS and ESRF are offering data-collection services without the attendance of the researcher who supplied the crystals (Bowler *et al.*, 2015 ▸; https://www.diamond.ac.uk/Instruments/Mx/I03/I03-Manual/Unattended-Data-Collections.html). This unattended data-collection mode results in diffraction information becoming available in ISPyB without intervention by the researcher. A flawless connection between crystallization and diffraction information becomes essential to cope with the amount of data that becomes available (Rao, 2020 ▸). The *IceBear* software package has been developed with this in mind. *IceBear* allows the collection of all of the relevant project information (including sequences; Fig. 2 ▸) as well as all of the important information of the crystallization experiments. Subsequently, the researcher can select and mark crystals for synchrotron data collection. The selected crystals are automatically given a unique sample name (Fig. 1 ▸), which is then used to collect all relevant information for that crystal when proceeding to the various steps of the structure-determination pipeline, including crystal treatment and data collection at the synchrotron. The metadata required for upload to ISPyB are therefore available in the *IceBear* database, and a metadata-exchange protocol has been implemented in *IceBear* such that when once one or more dewars filled with cryocooled crystals are ready to send to the synchrotron then the metadata can be transferred automatically to ISPyB. An important feature of this protocol is that the link to the ISPyB information for each sample that has physically been shipped is made available on the dedicated *IceBear* crystal page (Fig. 2 ▸) for this sample. Therefore, once the crystal has been used for data collection and the results of the data-processing pipelines are available in ISPyB then this information becomes easily accessible to the researchers through a link on the crystal page.

## 2. Results   

### 2.1. Design philosophy   

*IceBear* is a web-based application that is accessed through the user’s web browser. It is written in the PHP scripting language running on an Apache web server with a MySQL or MariaDB database running in the background. All of these components run on a low-cost Linux computer, with the latest Ubuntu Server LTS release being the Linux distribution of choice.

**Figure 3:**
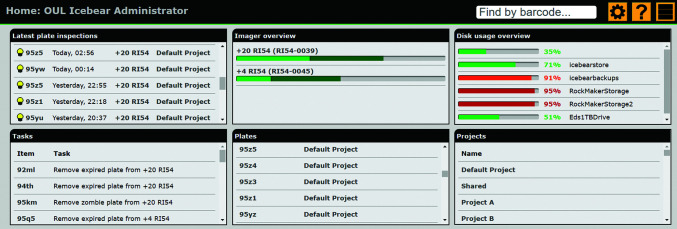
The *IceBear* home page. The ‘Latest plate inspections’ brick provides information on the latest imported plate inspections that the project owner should assign to projects. The ‘Tasks’ brick reminds the project owner of tasks that require attention. The ‘Imager overview’ brick visualizes the load on the imaging systems. Top right: the search box can be used to find relevant pages when searching for plate barcode, pin barcode, puck barcode, dewar barcode, sample name or PDB code. The wheel button allows the selection of preferred configurations for this page. The question mark button provides help for this page. The action button points to the page where various actions can be initiated (see text).

*IceBear* aims to empower researchers rather than getting in their way. The intent is to encourage researchers in a user-friendly way to provide all of the information as soon as possible, but not to insist until it becomes necessary. The overriding philosophy is to provide a clean and minimal interface focusing purely on the tasks at hand. The interface can be configured to the user’s preferences and working habits, including the layout and content of the home page (Fig. 3 ▸).

Among other means, users are encouraged to perform the required actions by highlighting actions needing attention and by means of a personalized task list on the researcher’s homepage. This approach extends beyond *IceBear* itself and includes, for example, adding an item to the user’s task list when their crystallization plates should be removed from the imager.

Flexible tools are available to record information when proceeding along the structure-determination pipeline. For example, the user can add notes and/or upload files with information. This is particularly relevant once a crystal has been selected and assigned a sample name by the system. Attaching files or notes (for example with links to electronic laboratory notebooks) as metadata associated with plates is also a good way of preserving upstream information, for example on the sample quality. Notes and files can be added by project members with write access, for example during crystal cryocooling, during data collection or later. Such notes and files are useful to inform other project members about the progress of the project. Notes also allow the sample to be linked to other electronic laboratory notebooks.

### 2.2. Installation and updates   

*IceBear* is designed to be installed by a reasonably proficient computer user with root permissions for the computer on which *IceBear* is installed. An *IceBear* installer has been developed. An initial install script configures the server and installs any necessary packages and PHP extensions, as well as configuring a basic firewall. The installation then continues in the web browser, where an administrator username and password, along with connection details for any on-site imaging hardware, are provided. An initial bulk import of users from the imaging system is also performed.

*IceBear* can be updated to a later version by any user with administrative privileges. This is performed via a dedicated user interface within the web application itself; no typing or command-line interaction is required in most cases.

The standard installation of *IceBear* can import images of crystallization drops generated by the Formulatrix (with Rock Maker user interface) and Rigaku (with CrystalTrak user interface) imaging equipment. When using the Formulatrix/Rock Maker setup the other crystallization metadata are provided by the project owner using a user-friendly GUI, as explained below. For the Rigaku/CrystalTrak setup the import of the other metadata can be performed in the same way, with small adjustments depending on the local situation. The software is available on request from the authors, as described on the *IceBear* website (https://icebear.fi/), and is distributed under an MIT license. The *IceBear* website provides extensive documentation and has a link to the demonstration server that allows hands-on practicing using the features and protocols implemented in *IceBear*. *IceBear* is installed at the protein crystallography research centers at the Universities of Oulu, Helsinki, Turku and Åbo Akademi in Finland and at the Weizmann Institute of Science in Israel.

### 2.3. Management in the home laboratory   

Smooth functioning of the *IceBear* installation requires that a member of the local protein crystallography community acts as an expert manager. Tasks include taking care of software updates as well as monitoring the disk space in use by both the live system and its backups. Ideally, this task should be allocated to someone who is highly familiar with the protocols involved in the daily operation of the crystallization facility. It will be important to train new *IceBear* users and point out the basic principles with respect to user rights and mode of operation. New users need to be assigned a username and appropriate rights. Several user categories exist. A special category of user (shippers) can coordinate the uploading to ISPyB of the metadata corresponding to the physical shipping of one or more dewars to a synchrotron. Shippers need to have access to the ISPyB ‘proposal’ of the data-collection session. A key category of user is the project owner, who is able to add new proteins and sequences to a project and who can also add researchers with their user rights to the project group. A third category of user (project members, with write access) can upload notes and files, and can select crystals. The basic category of user (project guests, with read access) can see all of the information on a project but cannot change it. The user management is flexible and user rights can easily be added, removed or transferred. An online user manual is available and context-sensitive help functionality has been integrated.

### 2.4. The *IceBear* home page   

The home page (Fig. 3 ▸) provides general information, for example concerning the load of the imagers, and also warnings on plates that are expired and should be removed from the imagers. The user is also warned if the crystallization information is not complete or if other metadata required for data collection at a synchrotron are not yet available. The home page is configurable; individual users can add, remove and rearrange elements to suit their needs and their way of working. For example, an administrator can add a graph of disk usage, enabling the disk space used by both *IceBear* and the imaging system to be monitored at a glance.

**Figure 4:**
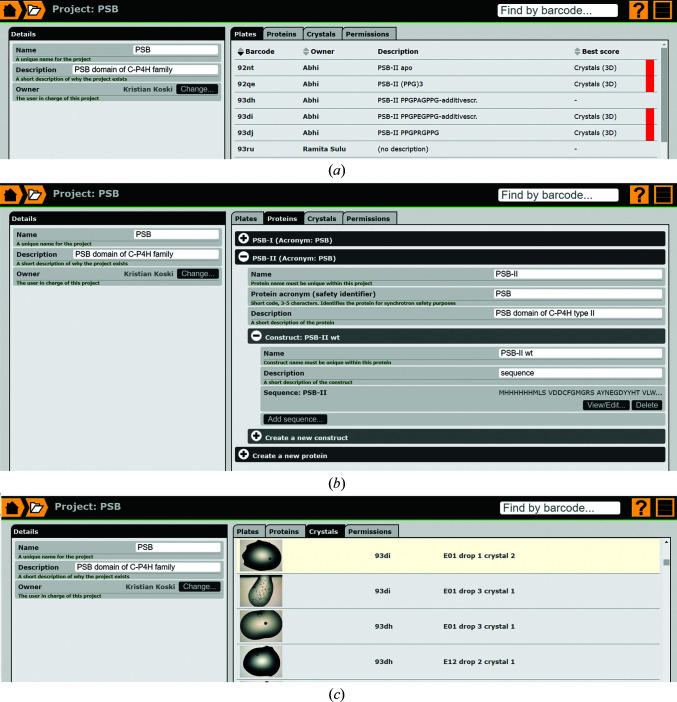
The project module. (*a*) The Plates tab lists all plates of this project (Murthy *et al.*, 2018 ▸). The ‘Description’ column provides a short description provided by the plate owner, which can be provided when the first inspection becomes available. The ‘Best score’ column is highlighted when a drop of this plate has been scored (Fig. 5 ▸) or if a crystal in a drop has been selected (Fig. 6 ▸) in the drop-viewer menu. (*b*) The Proteins tab provides information on the acronym, as well as a more complete protein name, and a description can be given. Each protein can have several constructs and the constructs can have multiple sequences. (*c*) The Crystals tab highlights all crystals selected from this plate.

As is discussed in the subsequent sections, the search box on the home page (top right in Fig. 3 ▸) and many other pages is a navigation tool to reach pages with the relevant information. Through the action button on the home page (top right) a page is opened that provides links to various actions, such as starting a new project, a new crystal-fishing session or a new shipment, as well as information on existing projects (Fig. 4 ▸). On this page, links are also provided to pages with information on screens and plate types, as well as information on containers (pins, pucks, dewars) known to *IceBear*.

### 2.5. Setting up a project   

The *IceBear* database is built around projects, referring to all of the work on a given protein or group of proteins. When setting up a project, a name and a short description are required (Fig. 4 ▸ *a*). After creating the project, at least one protein must be created inside it. When creating a protein, the project owner decides on an acronym that will be used for the safety sheet when collecting data at the synchrotron (called an A form at ESRF and ERA at DLS). The acronym is also used by the software when generating the sample name for a selected crystal, as will be discussed later. Within one project, several acronyms can be specified for different proteins. The case-sensitive acronym should be as short as possible (at most five characters is recommended) and should not contain special characters. Different projects can use the same acronym.

Each protein can have several sequence sets, referred to as constructs (for example a wild-type sequence and a point-mutated sequence). Each construct can have several sequences in case the studied protein is an assembly of multiple chains of different sequences. In most cases there will be one sequence, but in crystallization experiments with heterodimers (or heterooligomers in general) there can be more than one sequence in one set. It is possible to have several sequence sets in one project, for example when mutated variants (such as point-mutation variants) are being studied within the same project. It is also possible to have mutated variants in different projects (for example separate domains) but still use the same acronym. In general, it is good practice that the protein sequences of the proteins include tags that have been used, as is also required when depositing a structure in the PDB (https://www.wwpdb.org/documentation/policy). This sequence information can be provided either as the nucleotide sequence (which will then be translated into the amino-acid sequence) or as the amino-acid sequence. Setting up a project includes assigning write access (project members) and read access (project guests) to researchers that already have an *IceBear* account, such that small project groups can be formed to monitor the progress of the project. Project members with write access can upload notes and files and can select crystals for data collection. The project owner, project members and project guests can jointly monitor the available information of a project. The project page also provides a list of all of the crystals that have been selected (Fig. 4 ▸ *c*).

### 2.6. Setting up the crystallization plate, providing the metadata for each plate and importing and assigning a plate inspection to a project   

**Figure 5:**
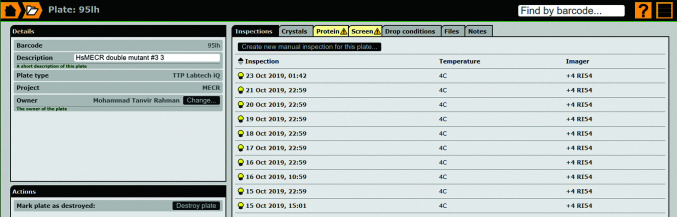
The plate-information page. The Inspections tab lists all available inspections of this plate. The Crystals tab lists all selected crystals (see also Fig. 4 ▸ *c*). The yellow triangle in the Protein tab (Supplementary Fig. S1*a*) and the Screen tab (Supplementary Fig. S1*b*) informs the plate owner that information on the protein buffer and the screen information, respectively, has not yet been provided. The Files and Notes tabs can be used to provide extra information relevant to this plate.

In the standard workflow, crystallization plates are prepared manually or by using a nanodispenser. These plates have the 96-well standard format and the crystallization drops are imaged by the automatic imaging system. The plates can have one drop or three drops per well (Supplementary Fig. S1*a*). When using plates with three drops, it is possible to use the same well solution for different protein concentrations, different ligands or different proteins. This information can be provided to *IceBear* in a user-friendly format. The information on screens can also be provided in several formats, including CSV, Rock Maker XML (https://formulatrix.com/life-science-automation-blog/rock-maker-xml-screen-updates/) and MIMER (Brodersen *et al.*, 2013 ▸) formats (Fig. 5 ▸, Supplementary Fig. S1*b*). Once the plate has been imaged, *IceBear* imports the plate information (identified by the plate barcode) from the imager into its database, sets its temperature and plate type, imports the images and assigns the plate to the researcher who has set it up based on the metadata provided by the imaging system. The *IceBear* software continuously checks whether the imaging systems have generated new images. In practice, any new image is imported with a 5 min delay. In the plate-information page (Fig. 5 ▸) it is also possible to make notes for this plate and upload files, which could be a file that contains the screen information or any other file with notes relevant to this plate (such as additives and ligands). The plate owner subsequently assigns a plate to a project by setting its protein and construct. The plate owner also provides the additional information on the plate, such as the crystallization screen, drop volumes and protein buffer (including protein concentration) used for each plate. This can be performed when the first plate inspection becomes available or later. Information on screens, as made by the researcher, can also be provided as simple two-column text files.

### 2.7. Monitoring the crystallization results and selecting crystals   

**Figure 6:**
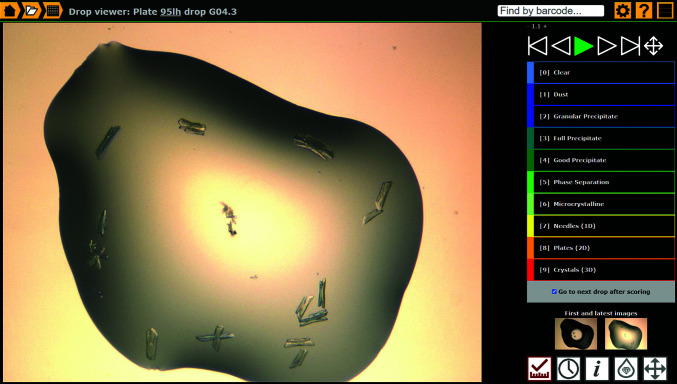
The drop-scoring page of the drop viewer. The drop viewer has five pages represented by the five icons at the bottom right. The drop-scoring page can be used to browse through the drops on a plate using the arrows at the top right (the arrow keys of the keyboard can also be used for browsing from one drop to another). A color code can be chosen to provide information on the contents of the drop. The other icons (bottom right) refer to the time lapse (Supplementary Fig. S2*a*), the crystallization conditions (Fig. 7 ▸ *a*), the crystal selection (Fig. 7 ▸ *b*) and the plate overview (Supplementary Fig. S2*b*).

**Figure 7:**
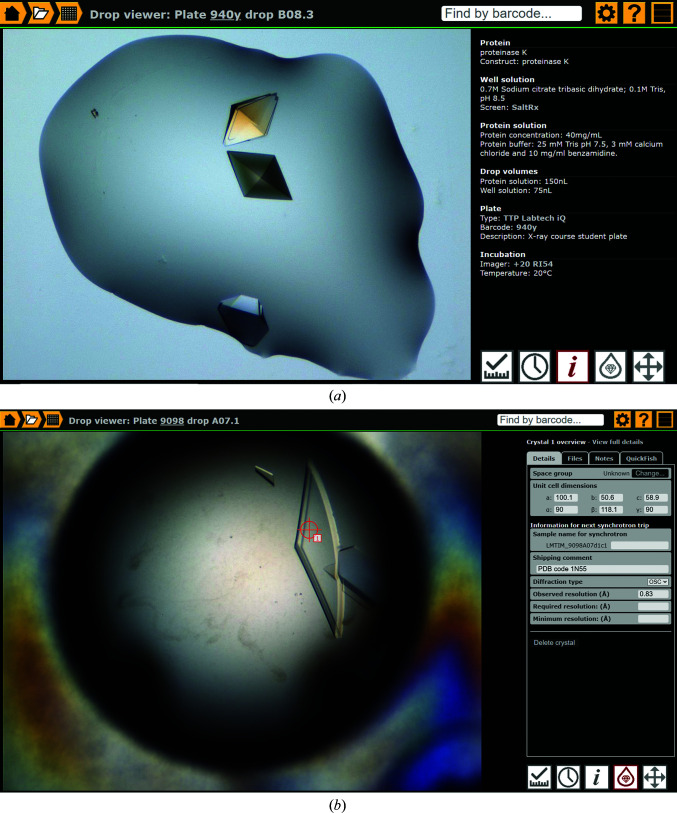
The drop viewer. (*a*) The crystallization conditions. The composition and pH of the well solution buffer and the protein buffer are provided. (*b*) The crystal-selection page. In this example a crystal has been selected and its sample name (LMTIM_9098A07d1c1) is provided, which is constructed from the sample acronym, the plate barcode, the well, the drop and the crystal number (see text). When a crystal has been selected then it is possible to extend the sample name that is generated by the system with a user-chosen extension, but the sample name should not be longer than 27 characters (see text). In addition, at this stage a crystal page is generated which can be reached by clicking ‘view full details’ (top right). The QuickFish option (top right) allows the crystal to be connected to a barcoded pin, independent from a scheduled synchrotron visit. Information for ISPyB can also be added at this stage in the right panel ‘Information for next synchrotron trip’.

A key module of the *IceBear* software is the drop viewer (Fig. 6 ▸). The pages of the drop viewer can be reached in several ways and it has five main functions (Fig. 6 ▸). (i) Measuring and scoring, allowing fast hands-free viewing of all images of a plate along with scoring and (where scale information is provided by the imager) measurement of the size of the crystal. (ii) Viewing the imaging history, where the change in the drop contents over time can be observed as a movie displaying the same drop as recorded in different inspections (Supplementary Fig. S2*a*). (iii) Showing crystallization conditions and other key information (Fig. 7 ▸ *a*). (iv) Crystal selection, allowing crystals to be marked for onward processing. (v) Navigation, showing a plate overview that can be toggled between visible and ultraviolet images, along with side-by-side comparison of all subpositions within a well (Supplementary Fig. S2*b*). Keyboard shortcuts throughout enable rapid interaction.

The crystals marked on the selection page are given unique sample names. The sample name is generated from the acronym extended by the plate barcode and the well, drop and crystal number (Fig. 7 ▸ *b*). The sample name can be extended by the user to make custom identification and subsequent navigation easier. For ISPyB the sample name can be at most 45 characters, but a maximal length of 27 characters is recommended as different synchrotrons have different implementations. It is possible to specify at this stage (or later) information on the space group, cell dimensions, known and desired resolution, which will be automatically uploaded during shipment to ISPyB. Notes can also be added to each crystal, now identified by its sample name, that has been selected on this page, as well as at various later stages of the project, for example at the crystal-treatment steps (such as cryocooling), during data collection and later at the crystal page, for example when refining the structure. Files, such as diffraction images, data-processing log files and scanned notes, can be attached to the crystal. A ‘quick-fish’ option is available at the crystal-selection page (Fig. 7 ▸ *b*) if the crystal is fished and mounted on a barcoded pin independent of shipment to a synchrotron, for example for data collection at a home source. This option links the crystal to a specific barcoded pin in the *IceBear* database. This pin plus crystal can be moved directly to the puck in the crystal-fishing module (or when finalizing the shipment) if synchrotron data collection is required.

### 2.8. The workbench for cryocooling crystals when using the crystal-fishing module of *IceBear*   

On the crystal-selection page, drops with suitable crystals can be identified in advance of the actual crystal-harvesting session. For harvesting and cryocooling the selected crystals, a crystal-mounting bench area is required equipped with a microscope for crystal viewing and sufficient space for preselected pins and pucks, as well as a computer with a barcode reader to read the barcodes of dewars, pucks, pins and plates. A high-quality barcode reader is recommended in order to ensure reliable reading of the small pin barcodes. The crystal-fishing GUI is optimized for use with a touch screen, but can also be used with a mouse. The computer should have access to the internet in order to run *IceBear* from the web browser and to connect to the synchrotron ISPyB website. Liquid nitrogen for crystal cryocooling and for storing pucks should also be available at the crystal-mounting bench. It is highly recommended to use barcoded pins, but the protocols also work with pins that have no barcodes (in which case the position of the pin in the puck is the sole means of data tracking).

### 2.9. The crystal-fishing module   

**Figure 8:**
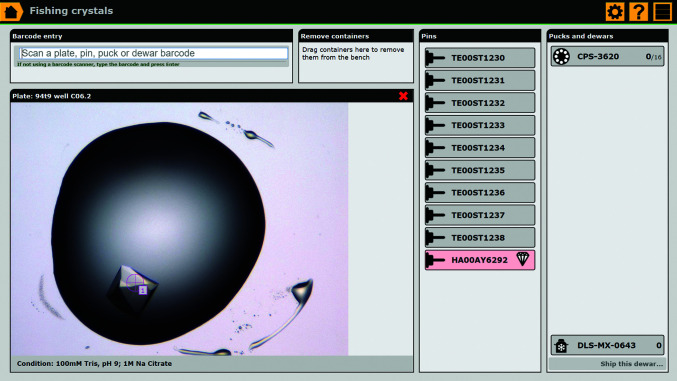
The crystal-fishing module. Fishing from a plate with preselected crystals. The barcodes of the pins, pucks and dewars are first scanned with the barcode reader. Once the plate barcode has been scanned then the drops with selected crystals are highlighted. The crystal is moved from the selected drop by a mouse movement or a touch-screen movement from well to pin to inform the database on the barcode of the pin that is associated with the fished crystal. The pin highlighted in red is already associated with a crystal.

The crystal-fishing GUI (Fig. 8 ▸) is designed to be used simultaneously with the cryocooling of crystals. This GUI associates cryocooled crystals with a pin barcode and with a numbered position within a puck. The use of non-barcoded pins is also supported, in which case data tracking relies completely on the position of the pin in the puck. The crystal-fishing GUI aims to reduce the required interaction with the GUI to an absolute minimum when cryocooling crystals, with large draggable elements optimized for use with a touch screen. In this way, crystal fishing and updating the *IceBear* database can be performed simultaneously. The GUI works as a virtual workbench. Plates as well as containers, such as pins, pucks and dewars, are added to the virtual workbench by scanning their barcodes. A crystal can then be moved to a pin merely by dragging and dropping; by default, the pin is then added automatically to the first empty position of the first puck, but it is possible to move a pin to another position in the puck at this stage or at a later stage. The sample name of the first crystal that is fished from a drop will be referred to as crystal 1 (c1), and subsequently fished crystals will be referred to as c2 *etc*. For non-barcoded pins, dragging the crystal directly to the puck will create a dummy pin. If a barcoded pin already contains a crystal, *IceBear* knows this and a ‘crystal’ symbol appears with the barcode (Fig. 8 ▸). Notes can be added immediately after fishing, with one-touch buttons available for common notes, but this is not needed to complete the fishing action. For repetitive treatment actions, recent notes are available for reuse. User-specific features of this protocol can be selected by the user in the configuration options of this page.

The crystal-fishing GUI is designed to very easily, with minimum distraction, connect preselected crystals to barcoded pins, pucks and dewars when crystals are fished from 96-well plates which have been imaged before and which have preselected crystals. However, the GUI can also be used if the home laboratory does not have a crystallization-drop imaging system or if the plate has not been imaged before. In this case, the crystal-fishing GUI creates a dummy image as well as a unique sample name. The GUI can also be used if the crystals have been cryoprotected after an extensive crystal-treatment protocol for ligand-binding studies (Hassell *et al.*, 2007 ▸). For example, the crystals are incubated for different time spans in drops of different pH, different precipitant or different ligands at different concentrations and consequently cryoprotection is therefore performed at different time points. The information on these crystal-treatment steps can be documented in notes and the same crystal-fishing GUI can be used in the same way to inform the database which sample has been cryocooled, on which pin, in which puck and in which dewar, at a convenient time before the metadata of the shipment are uploaded to ISPyB.

The data-tracking options of *IceBear* are optimally exploited when using automatic crystallization-drop imaging devices, but their use is not strictly necessary. In any case users can also image the drops on their plates manually, and either upload microscope images if available or work without adding any drop images to *IceBear*.

### 2.10. The shipment module and the shipper   

**Figure 9:**
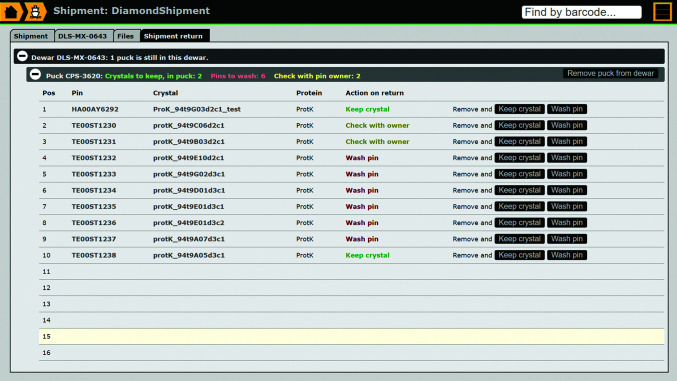
The dewar return page of the shipment module. This page facilitates the recycling of the pins, pucks and dewars when the dewar returns to the laboratory. Washed pins will be available for picking up new crystals for the next shipments. The pins highlighted as ‘Keep crystal’ will be placed in a storage dewar by the researcher and are then available for the next synchrotron trip.

The *IceBear* shipment interface (Supplementary Fig. S3) is designed for assembling shipments and submitting them to synchrotrons. Dewars filled with samples can be brought directly from the crystal-fishing interface, but dewars, pucks and pins that have been prepared previously can be added or removed prior to shipping, for example if a pin or a puck is brought from storage to fill an empty slot in a puck or dewar, respectively, at the last minute. Typically, a dewar shipment to the synchrotron will contain crystals from several research groups, which all have crystals that belong to the same proposal and that are participating in the same data-collection session. In order to ensure that the uploading of the metadata proceeds smoothly, this is coordinated by an *IceBear* shipper, who is also registered at the synchrotron. In practice, the *IceBear* shipper organizes the shipment and coordinates the activities when the dewar returns (Fig. 9 ▸).

Where supported by the synchrotron, *IceBear* can submit the shipment metadata directly to the IT systems at the synchrotron. Currently, this is supported for shipments to DLS using the SynchWeb/ISPyB application programming interface (API; Fisher *et al.*, 2015 ▸). In this case the metadata of the shipment are uploaded by the *IceBear* shipper, who is part of the synchrotron proposal and the synchrotron data-collection session. The shipper authenticates with the synchrotron and then selects the proposal and session from those he/she is authorized to use, before submitting the metadata of the shipment. No typing is required other than the synchrotron username and password. All container and sample details are transferred automatically to the synchrotron, with no manual data entry at the synchrotron site needed. *IceBear* performs pre-upload validation on its own shipment data, ensuring that for example no empty containers (pins, pucks or dewars) are submitted. Crucially, the shipment will not be submitted if the ‘protein acronym’ safety identifier of any crystal is missing from the approved list of acronyms for this shipment or if any crystal does not have a protein acronym assigned. After completion of the uploading of the metadata to DLS, the SynchWeb/ISPyB tools for tracking the dewar shipments by the courier services are then used before the dewars are sent.

A shipment manifest in PDF format (Supplementary Fig. S4) is generated when the shipment is submitted. The format allows freehand note-taking on the printed version during data collection if desired. For synchrotrons where automated uploading of metadata is not supported, such a shipping manifest is also generated. This manifest contains clickable links and scannable QR codes to both *IceBear* and ISPyB. The *IceBear* link points to the crystal page and the ISPyB link points to the page where the ISPyB information on that crystal is provided.

### 2.11. Making notes during the data collection   

Once the shipment has been marked in *IceBear* as sent, a new Data Collection tab is made available in the shipment GUI. Here, every puck in the shipment is shown as a clickable button. Clicking a puck opens a set of tabs (Supplementary Fig. S5), one per pin position. During data collection, it is possible to mark that data have been collected from the crystal at this position. It is also possible to add notes for each sample name. Some standard note template texts are available as one-click buttons. The user can also specify here whether the crystal should be kept or washed/discarded on return to the home laboratory. Pins marked as being used for data collection and pucks, where all pins are marked as being used, are shown in green with a check mark.

### 2.12. Recycling the pins, pucks and dewars when the dewar returns to the laboratory   

When the dewar is returned to the home laboratory, the shipper can then mark in *IceBear* that the dewar has returned. The Data Collection tab of the shipment GUI (Supplementary Fig. S5) is then replaced by a Shipment Return tab (Fig. 9 ▸). The shipper can then unpack the dewar(s). The Shipment Return tab shows the contents of each dewar and puck, with the crystal owner’s specified action, *i.e.* ‘Keep crystal’ or ‘Wash pin’, shown for each sample in the shipment. The researchers with samples in this shipment clean their pins or store their pins and crystals for later use. Unpacking/washing the containers (dewars/pucks/pins) in *IceBear* makes them available for reuse in subsequent shipments.

### 2.13. The crystal page and other navigation tools   

**Figure 10:**
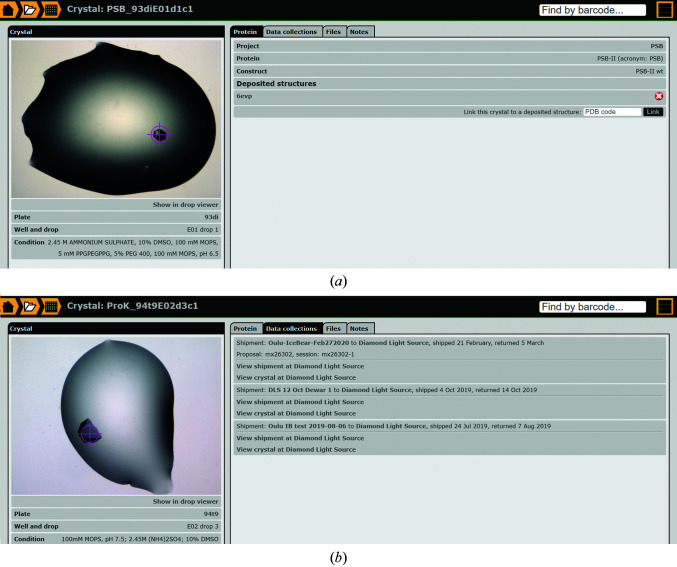
The crystal page. (*a*) The Protein tab. Key information available for this sample is provided on this page. It is possible to navigate to this crystal page by putting the sample name ‘PSB_93diE01d1c1’ in the search box. In this case a PDB deposition has been completed with PDB code 6evp (Murthy *et al.*, 2018 ▸). It is possible to provide multiple PDB codes. The red cross is the undo button (in case the wrong PDB code has been given). The link ‘Show in drop viewer’ points to the drop viewer and provides access to information on the crystallization conditions. (*b*) Data-collection information. This crystal has been used as a test crystal on three different rapid-access data-collection sessions at DLS using beamline I03. The link ‘View crystal at Diamond Light Source’ points to the relevant ISPyB page for this crystal.

The crystal page (Fig. 10 ▸ *a*) is generated for every crystal that has been selected (in the selection menu of the drop viewer; Fig. 7 ▸ *b*) and for which therefore a unique sample name has been generated. This page also records the information on data-collection sessions at the synchrotron and provides their proposal ID and session ID. The same crystal can be shipped multiple times to synchrotrons (Fig. 10 ▸ *b*). The crystal page is reached from the shipment manifest, but can also be found when typing the sample name in the search box (Fig. 3 ▸). From the crystal page there is a link to the diffraction information in ISPyB (Fig. 10 ▸ *b*) as well as to the drop viewer (Fig. 7 ▸), and therefore to the project information and the crystallization conditions. The crystal page can also be reached from the crystal-selection page of the drop viewer (Fig. 7 ▸ *b*). If a structure has been deposited in the PDB (Adams *et al.*, 2019 ▸) then the PDB code can be provided on this page (Fig. 10 ▸ *a*), and the crystal page can then also be found via the search box by specifying the PDB code.

## 3. Discussion   

### 3.1. The rapid-access beam-time data-collection sessions at DLS   

The described protocols have been tested and optimized in several data-collection sessions at DLS (Fig. 10 ▸ *b*). The optimal use of the developed system requires some extra work from the expert user. For example the details of every project (sequence information, acronym) have to be provided and also relevant information on every plate (the plate type, the screen that has been used, the drop conditions, the protein buffer) should routinely be provided. With respect to cryocooling, it is very helpful for the expert user as well as for the novice user that only highly relevant information is requested and this information does not have to be provided immediately. In general, it is very important to have easy access to all important experimental information on crystallization and crystal treatment for later use and also for others to be able to reproduce the experiments. It is also beneficial that information is easily shared between project members. Another practical advantage is the dewar management. In *IceBear*, protocols have been implemented to recycle the dewar contents once the dewar is back in the home laboratory, which make it easier to keep track of the pins, pucks and dewars that are in use in the laboratory. Via the crystal page, the proposal ID and session ID (Fig. 10 ▸ *b*) used for data collection from a specific sample are provided. Most importantly, it now becomes transparent to check the data-collection details (available in ISPyB) when refining the structure. This allows the user to go back and check whether there are ice rings, whether there is a space-group ambiguity or radiation damage, anisotropy, resolution or any other issue recorded by the automated data-processing pipeline that warrants a more detailed inspection of the data-collection information.

### 3.2. Navigation options   

Extensive navigation options are available to find the relevant information. The search box can be used to search for plates, pins, pucks and dewars using their barcodes, and is frequently used during the crystallization and data-collection stage of a project. A key identifier is the sample name: all of the information on a particular crystal can be found using the sample name, which can also be used as a search item in the search box. From the crystal page a link to the relevant ISPyB page is available, as well as a link to the drop viewer, where the crystallization conditions can be found. *Vice versa*, from the crystal selection page of the drop viewer it is possible to reach the crystal page, which will have information on whether the sample has been sent to the synchrotron; if this is the case, and if diffraction information has been collected, then the link to the ISPyB diffraction information is also available from this page. After data collection at a synchrotron the crystal page can also be reached using the link for a particular crystal, as provided in the shipment manifest (Supplementary Fig. S4).

Good navigation options are also important in later stages of a project, for example when refining the structures and when writing a manuscript. It is not atypical for this writing to take place several years after crystallization and data collection have been carried out. For data tracking at this stage of the project it is important to find the relevant crystal page, which can be found using the search box and specifying the sample name (which is also available on the ISPyB page) or the PDB code. If the sample name has been extended by a user-chosen character set (for example a ligand name) then it is possible to find the relevant crystal pages for that ligand via the search box by specifying only the relevant ligand name. From the crystal page, the project page can also be found using the information on the construct(s) used.

### 3.3. Data tracking   

The relevant *IceBear* crystal page can be found using the PDB code. This makes it easy to find the diffraction information in ISPyB as well as the crystallization and crystal-treatment information associated with a PDB code. On the crystal page, notes and files can be uploaded for the respective sample. If the researcher has archived the raw data used for data processing in an open-access archiving system, then the DOI and other relevant information can be documented using the notes and files options available on the relevant crystal page. Similarly, links to relevant publications can be stored in the database and made available to the project members. The notes and files option can also be used to document intermediate results, for example when a project member leaves the project when the structure has not yet been deposited.

### 3.4. An educational tool   

*IceBear* is organized around projects. In a university setting a project will typically consist of student, direct supervisor, senior supervisor and other group members as well as on-campus and off-campus collaborators. *IceBear* provides the IT tools to facilitate expert discussions about the progress of the project with respect to (i) the crystallization experiments as well as, importantly, (ii) the diffraction properties of the harvested crystals, including the diffraction data quality, as can be deduced from the log files of the data-processing pipelines. These tools allow the optimal use of the available human and hardware resources both at the home laboratory as well as at the synchrotron.

## 4. Concluding remarks   

Protein crystallography is expected to remain an important method for understanding what determines the specificity and affinity properties of proteins and therefore for understanding the relationship between their structure and their function (Grimes *et al.*, 2018 ▸; Förster & Schulze-Briese, 2019 ▸) and for translating this knowledge into applications (Malbet-Monaco *et al.*, 2013 ▸; Materlik *et al.*, 2015 ▸). Therefore, the unique properties of the X-ray beamlines of synchrotrons (Winter *et al.*, 2019 ▸) remain of strategic importance in order to be able to proceed efficiently with all of the steps of structure determination. The vast majority of crystallization experiments are carried out in home laboratories situated far away from the synchrotron X-ray source. Therefore, efficient exchange of the crystal metadata between the home laboratory and synchrotron, as implemented in the *IceBear* software (Fig. 1 ▸), is of critical importance. This now makes it easy to address questions related to data collection and data processing of a sample when refining its structure and to correlate this information with the experimental details of the crystallization and crystal-handling experiments (Fig. 10 ▸, Supplementary Fig. S4). Future versions of *IceBear* are being considered which include the implementation of scoring protocols when importing the crystallization-drop image, allowing the presence/absence of crystals (Bruno *et al.*, 2018 ▸) or the presence/absence of promising microcrystals/versus denatured protein (Ng *et al.*, 2014 ▸) to be predicted. Hosting *IceBear* as a cloud service, which is currently being tested using the CSC (IT Center for Science at Espoo, Finland), could help in the implementation of *IceBear* installations in new laboratories and will also provide computing power for improving automated scoring procedures. Other improvements that are planned, for example to cope with large number of data sets, concern importing diffraction-quality indicators from ISPyB (such as resolution) followed by ranking, for example per plate or per project. Such tools will facilitate extensive fragment-screening campaigns. For the implementation of database queries it will be important that the compound nomenclature is standardized (Lynch *et al.*, 2020 ▸; Abrahams & Newman, 2019 ▸).

The *IceBear*/ISPyB metadata-exchange protocol is currently available for the SynchWeb/ISPyB system (Fisher *et al.*, 2015 ▸) as implemented at DLS. The other European synchrotrons have implemented different flavors of ISPyB and collaborations have been initiated to provide this automated shipment option for multiple synchrotrons. In the future, it is planned to include the uploading of sequence, structure and ligand information of each sample to ISPyB, as the full power of the data-processing and structure-determination pipelines can then be exploited automatically.

## Supplementary Material

Supplementary Figures. DOI: 10.1107/S2059798320015223/nj5301sup1.pdf
