# Supplementary material for: IceBear: an intuitive and versatile web application for research-data tracking from crystallization experiment to PDB deposition
Source: Acta Crystallogr D Struct Biol. 2021 Jan 26;77(Pt 2):151–63. doi: 10.1107/S2059798320015223 (PMC7869904; doi:10.1107/S2059798320015223)
Supplement: Supplementary file 1 [file d-77-00151-sup1.pdf]

Acta Cryst D

STRUCTURAL BIOLOGY

Volume 77 (2021)

Supporting information for article:

IceBear: an intuitive and versatile web application for research-data tracking from crystallization experiment to PDB deposition

Ed Daniel, Mirko M. Maksimainen, Neil Smith, Ville Ratas, Ekaterina Biterova, Sudarshan N. Murthy, M. Tanvir Rahman, Tiila-Riikka Kiema, Shruthi Sridhar, Gabriele Cordara, Subhadra Dalwani, Rajaram Venkatesan, Jaime Prilusky, Orly Dym, Lari Lehtiö, M. Kristian Koski, Alun W. Ashton, Joel L. Sussman and Rik K. Wierenga

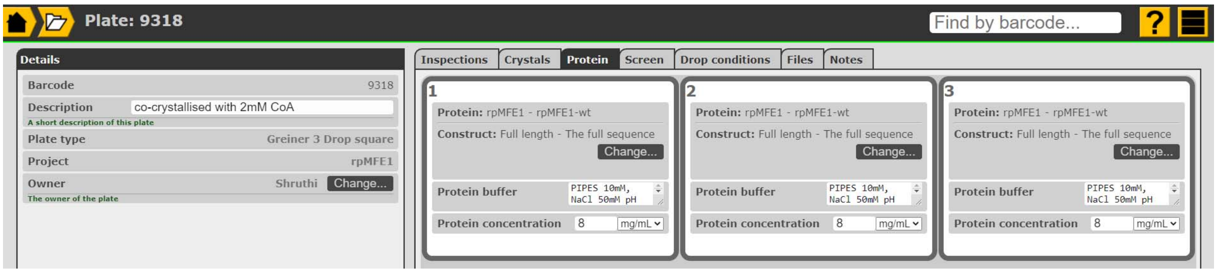

(*a*)

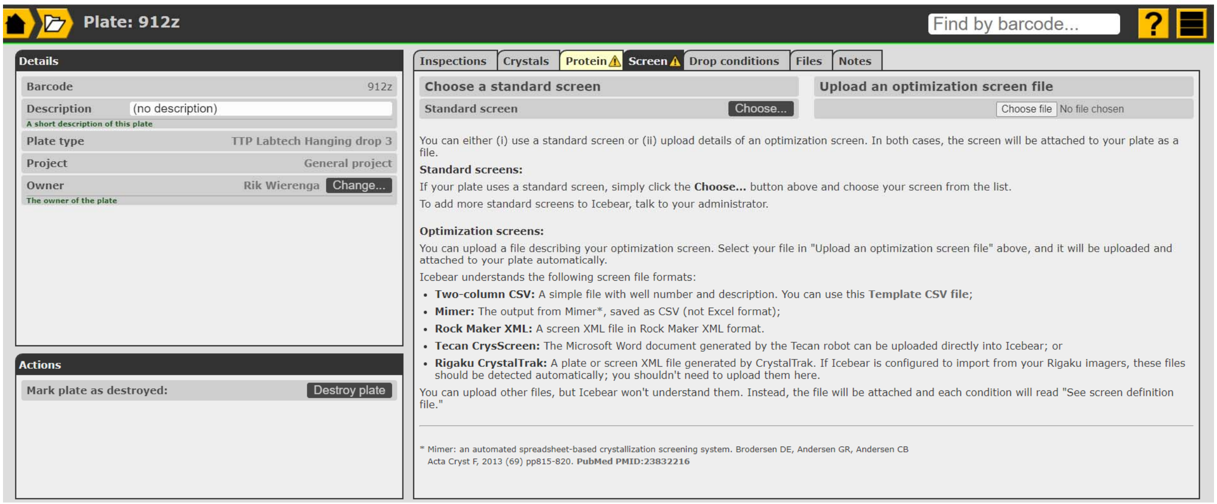

(*b*)

Figure S1 The Protein tab and the Screen tab of the Plate information page. (a) The Protein tab provides information on the protein buffer(s) used for the experiment. In case of multiple drops per well it is possible to have different proteins in the respective drops. (b) The Screen tab. This tab visualizes that the screen information is not yet provided and it allows the project owner to provide this information for this plate (912z) in various ways.

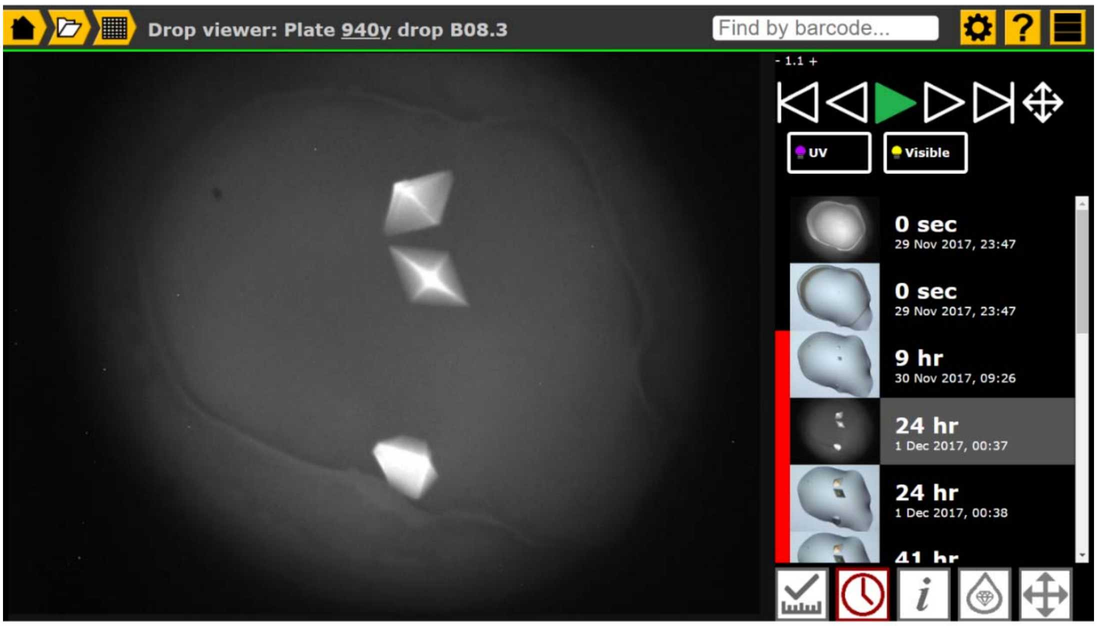

(*a*)

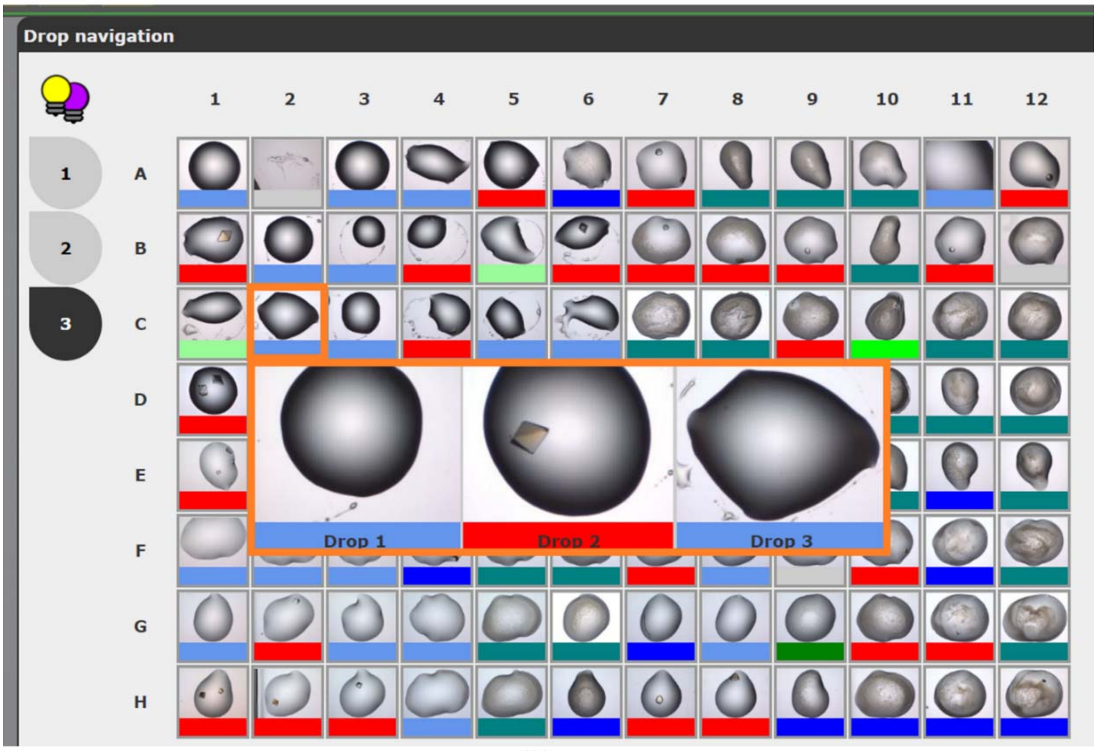

(*b*)

Figure S2 The time lapse and plate overview pages of the drop viewer. (a) The time lapse page displays all available inspections with a user chosen speed (top right, the value +1.1 can be changed to slow down or increase the speed). In this example, the UV images are shown. (b) The overview of this plate refers to drop 3 (as highlighted top left). It concerns the result of the visible light inspection. The color code visualizes the scoring results as chosen in the drop viewer menu (for example the red color indicates that the drop contains crystals). For drop C02 (chosen by where the cursor is) the results of drop-1, drop-2 and drop-3 are visualized.

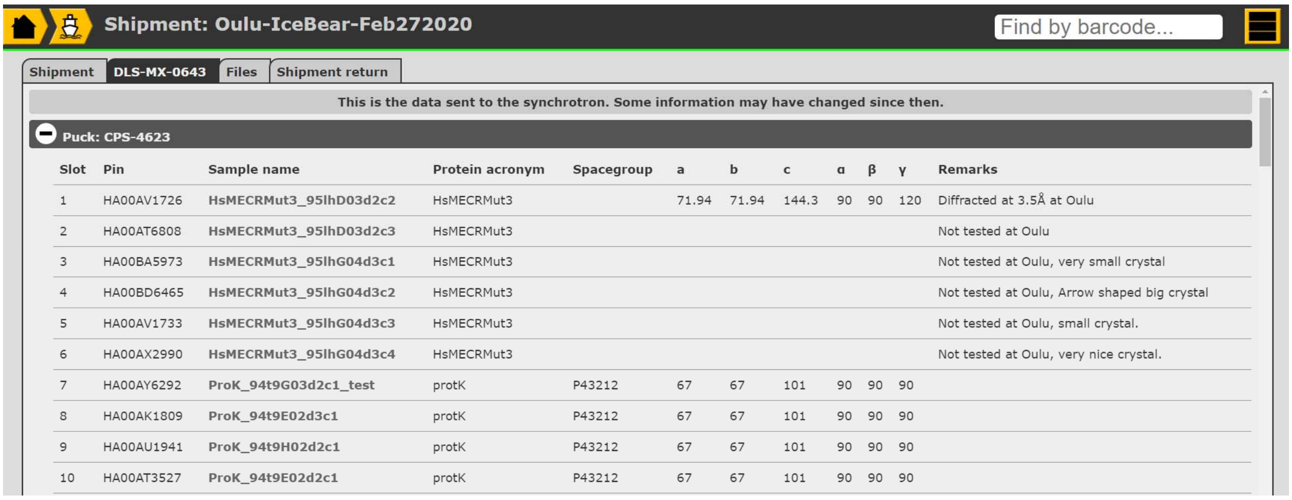

Slot	Pin	Sample name	Protein acronym	Spacegroup	a	b	c	α	β	γ	Remarks
1	HA00AV1726	HsMECRMut3_95lhD03d2c2	HsMECRMut3		71.94	71.94	144.3	90	90	120	Diffracted at 3.5Å at Oulu
2	HA00AT6808	HsMECRMut3_95lhD03d2c3	HsMECRMut3								Not tested at Oulu
3	HA00BA5973	HsMECRMut3_95lhG04d3c1	HsMECRMut3								Not tested at Oulu, very small crystal
4	HA00BD6465	HsMECRMut3_95lhG04d3c2	HsMECRMut3								Not tested at Oulu, Arrow shaped big crystal
5	HA00AV1733	HsMECRMut3_95lhG04d3c3	HsMECRMut3								Not tested at Oulu, small crystal.
6	HA00AX2990	HsMECRMut3_95lhG04d3c4	HsMECRMut3								Not tested at Oulu, very nice crystal.
7	HA00AY6292	ProK_94t9G03d2c1_test	protK	P43212	67	67	101	90	90	90	
8	HA00AK1809	ProK_94t9E02d3c1	protK	P43212	67	67	101	90	90	90	
9	HA00AU1941	ProK_94t9H02d2c1	protK	P43212	67	67	101	90	90	90	
10	HA00AT3527	ProK_94t9E02d2c1	protK	P43212	67	67	101	90	90	90	

Figure S3 The shipment information tab of the shipment module. The information of each sample listed in this figure present in dewar DLS-MX-0643 is available for a shipment that has been sent successfully to DLS. The metadata on space group, cell dimensions and remarks can be updated when finalizing the shipment for uploading. The space group information, cell dimensions can be added also on the crystal selection page as well as when fishing crystals. The file tab has a link to the shipment manifest PDF file (Figure S4). The shipment return tab provides information on actions to be taken when the dewar comes back in the home lab (see Figure 9).

Dewar DLS-MX-0643, puck CPS-4623

1	HA00AV1726	HsMECRMut3_95lhD03d2c2		
	Icebear	Diffracted at 3.5Ã… at Oulu		*Diamond Light Source*
2	HA00AT6808	HsMECRMut3_95lhD03d2c3		
	Icebear	Not tested at Oulu		*Diamond Light Source*
3	HA00BA5973	HsMECRMut3_95lhG04d3c1		
	Icebear	Not tested at Oulu, very small crystal		*Diamond Light Source*
4	HA00BD6465	HsMECRMut3_95lhG04d3c2		
	Icebear	Not tested at Oulu, Arrow shaped big crystal		*Diamond Light Source*

Figure S4 The shipping manifest. This PDF file is available from the Files tab of the shipment module. The PDF file is generated on completion of a shipment. It is generated also when the metadata is not uploaded to the synchrotron ISPyB. The link on the left side points to the IceBear crystal page of the sample name. The link on the right side points to the ISPyB page (at the synchrotron) of the sample name.

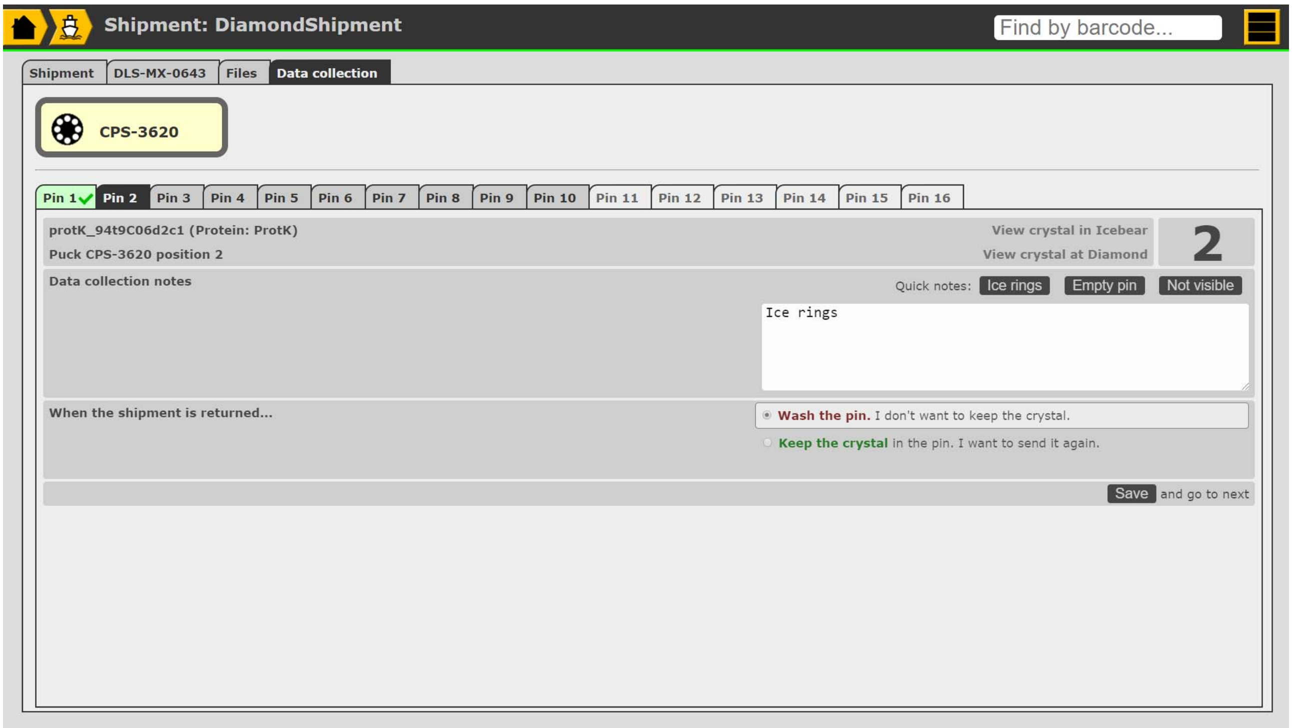

Figure S5 The data collection tab of the shipment module. During data collection it is possible to make notes for each crystal, as identified by its sample name. The "View crystal in IceBear" link opens the selection page of the IceBear drop viewer of this sample. The "View crystal at Diamond" link opens the ISPyB page of that sample. The researcher can provide the preferred treatment of the pin when the dewar returns back to the laboratory: by default, the pin is washed and becomes available for the next shipment, but it is also possible to activate the "Keep the crystal" preferred treatment. .
